# Implementation challenges in delivering team-based care (‘TEAMcare’) for patients with chronic obstructive pulmonary disease in a public hospital setting: a mixed methods approach

**DOI:** 10.1186/s12913-016-1592-2

**Published:** 2016-08-03

**Authors:** Belinda Cochrane, Jann Foster, Robert Boyd, Evan Atlantis

**Affiliations:** 1 School of Medicine, Western Sydney University, Penrith, NSW Australia; 2 Department of Respiratory and Sleep Medicine, Macarthur Health, Campbelltown, NSW Australia; 3 School of Nursing and Midwifery, Western Sydney University, Penrith, NSW Australia; 4 Discipline of Obstetrics, Gynaecology and Neonatology, Sydney Medical School/Sydney Nursing School, University of Sydney, Sydney, NSW Australia; 5 Ingham Institute, Liverpool, NSW Australia; 6 South Western Sydney Primary Health Network, Campbelltown, NSW Australia; 7 School of Medicine, The University of Adelaide, Adelaide, SA Australia

**Keywords:** Clinical practice guideline, Clinical protocols, Comorbidity, Multidisciplinary communication, Pulmonary disease, chronic obstructive (COPD)

## Abstract

**Background:**

Chronic obstructive pulmonary disease (COPD) is considered a multisystem disease, in which comorbidities feature prominently. COPD guidelines recommend holistic assessment and management of relevant comorbid diseases but there is limited information as to how this is best achieved. This pilot study aimed to explore the views of stakeholders, including patients and the healthcare team, on the feasibility, acceptability and barriers to a collaborative, multidisciplinary team-based care intervention (‘TEAMcare’) to improve health outcomes in COPD patients, within the context of a local hospital outpatient clinic.

**Methods:**

A mixed methods study design was used. A COPD care algorithm was developed based on the Australasian guidelines, COPDX. COPD participants were consecutively recruited from an outer metropolitan hospital’s respiratory clinic. Participants attended for follow up visits at 5 and 10 months to ascertain clinical status, algorithm compliance and to review and revise management recommendations. The intervention was conducted using existing resources, involving collaboration with general practice and the publicly-funded local chronic disease management programme (Medicare Local). Stakeholders provided qualitative feedback about the intervention in terms of feasibility, acceptability and barriers via structured and semi-structured interviews. All interviews were recorded, transcribed verbatim and analysed using qualitative thematic analysis to identify key concepts and themes.

**Results:**

The study protocol was abandoned prematurely due to clear lack of feasibility. Of 12 participants, 4 withdrew and none completed pulmonary rehabilitation (PR). The main reasons for non-participation or study withdrawal related to reluctance to attend PR (6 of 16) and the burden of increased appointments (4 of 16). PR conflicted with employment hours, which presented problems for some participants. Similarly, themes that emerged from qualitative data indicate healthcare provider perception of deficiencies in funding (for infrastructure and staffing). Health literacy, motivation, organisation and functional impairment were issues for patients.

**Conclusions:**

Available data from this small pilot provided valuable insights to inform future design and implementation strategies. Delivering structured team-based care to COPD patients presents challenges. In addition to enhancing health resources for engaging COPD patients, a focus on health literacy and improving health service access, including colocalisation and access outside business hours, may be required.

**Trial Registration:**

ACTRN12616000342415; 16/03/2016.

**Electronic supplementary material:**

The online version of this article (doi:10.1186/s12913-016-1592-2) contains supplementary material, which is available to authorized users.

## Background

Chronic obstructive pulmonary disease (COPD) is a leading cause of disability and early death [1]. In 2006, in Australians aged 40 years or more, it was estimated that the prevalence of COPD, defined according to the Global Initiative for Chronic Obstructive Lung Disease (GOLD) criteria as at least stage 2 (disease severity likely to be associated with significant symptom burden), was 9 % in men and 12 % in women [2]. This estimate increased markedly with age, independent of smoking history. COPD is a chronic disease and as disease advances so does the burden of comorbid illnesses, which interact adversely with the COPD pathophysiology and serve to complicate management in individual patients. Since Australia’s population is ageing, the projected increase in COPD prevalence will undoubtedly cause significant burden on the national healthcare system. Effective treatment and management of COPD and prevention of exacerbations would yield substantial health and economic benefits.

The COPD-X Plan – Version 2.43 represents the current Australian and New Zealand management guidelines for COPD [3]. These guidelines aim to “effect changes in clinical practice based on sound evidence; and shift the emphasis from a predominant reliance on pharmacological treatment of COPD to a range of interventions which include patient education, self-management of exacerbations and pulmonary rehabilitation” (page 10). However, published research before and subsequent to publication of the first COPD-X Plan suggests that conformity to COPD guidelines in Australia is actually quite limited [4]. The explanation for this lack of guideline implementation is likely to be multifactorial; the range and complexity of common diseases that present alongside COPD, and require coordinated multidisciplinary care plans, and also the limited availability and access to pulmonary rehabilitation (PR) services are considerations. For instance, in 2013 the Australian Institute of Health and Welfare (AIHW) reported that only five to ten percent of patients with moderate to severe COPD had accessed PR [5]. Moreover, in September 2015 there were only approximately 275 listed centres delivering PR programmes on Lung Foundation Australia’s website (http://lungfoundation.com.au) [6], mainly in urban and larger regional centres throughout Australia, expected to service an estimated 819, 311 Australians with moderate to severe COPD (GOLD stage 2–4); with estimates based on 2012 Australian census data, burden of obstructive lung disease (BOLD) study data and projections of population increase [7, 8]. Hence, awareness of PR programmes is generally low amongst COPD patients and programme access is mainly limited to urban-dwelling patients, who have had contact with a specialist respiratory healthcare provider. Importantly, there is currently no national funding mechanism for PR, although Medicare (Australia’s federally-funded universal healthcare scheme) provides up to five individual allied health services per calendar year available under Medicare for patients with a chronic medical condition and complex care needs, which theoretically could be used for physiotherapy sessions (to develop an exercise programme similar to that used in PR).

According to the COPDX Guidelines [3], comorbidities that should be considered routinely and treated appropriately in any patient with COPD, include nicotine dependence, common mental health disorders (depression and anxiety), cardiovascular disease, metabolic syndrome, skeletal muscle dysfunction, osteoporosis, lung cancer, and hypogonadism. COPDX establishes standards of care for COPD pulmonary disease, PR, and recommendations about assessment and management of comorbidities. However, there is little information about how this coordinated, collaborative care for COPD patients can be achieved in practice. Initiatives exploring telemedicine, case coordination and home-based PR [9–13] and various combinations have reported inconsistent results and no resounding success, in comparison with the definite beneficial effects of standard PR [14]. Since the guidelines include recommendations to implement multidisciplinary care plans for COPD based on limited evidence (NHMRC evidence level III-2), more trials are needed to inform clinical practice about the organization and delivery of multidisciplinary care for COPD patients within the Australian healthcare system. We aimed to explore the views of stakeholders including patients and the healthcare team on the feasibility, acceptability and barriers to a guideline-driven collaborative, multidisciplinary team-based care intervention (TEAMcare) to improve health outcomes in COPD patients within an outer metropolitan hospital’s respiratory clinic.

## Methods

The study used a mixed methods design. Mixed methods design allows the integration of quantitative and qualitative research approaches for the purposes of understanding the complexity of the problem [15]. In view of this, the term participants is used to highlight the importance of individual perspectives. The study protocol was approved and monitored by the Research and Ethics Office, South Western Sydney Local Health District (SWSLHD); Reference: HREC/12/LPOOL/349. Written informed consent for participation in the study, including agreement that non-identifiable data may be published, was obtained.

### TEAM care intervention and study population

A COPD care algorithm was developed based on the Australasian guidelines, COPDX [3]. COPD participants were consecutively recruited from an outer metropolitan hospital’s respiratory clinic. Participants were older than 40 years and demonstrated clinical features and spirometry criteria (Global Initiative for Chronic Obstructive Lung Disease, “GOLD” criteria) consistent with the diagnosis of COPD for eligibility. Exclusion criteria were applied; inability to walk safely (walking aids allowable), current participation in PR, unable to communicate in English, respiratory failure necessitating domiciliary oxygen, recent COPD exacerbation, unstable cardiovascular disease, acute illness and poor prognosis for long term survival. Participants gave informed consent prior to study participation and then underwent clinical assessment, with collection of demographic and clinical data.

For each participant, an individualised, integrated, multidisciplinary assessment and treatment plan for COPD was formulated and tailored to needs identified during the initial assessment, which included a risk assessment for various comorbid diseases associated with COPD. These individualised COPD management plans were based on the COPD care algorithm (Additional file 1) and typically comprised assessment of patient needs, establishment of management goals, disease-specific education and implementation of self-management strategies via coordination of treatment services by the allied health staff on the treating team, proactive monitoring and review. Case management was undertaken (via telephone and email liaison) for each participat to facilitate appointments for clinical investigations, clinician assessments and enrolment to PR. Participants attended for respiratory clinic follow up visits at five and ten months to ascertain clinical status, algorithm compliance and to review and revise their recommended management plan (Additional file 2). The intervention was conducted using existing resources and facilities and involved collaboration with the community’s general practices and the South Western Sydney Medicare Local to access the chronic disease management and prevention programme (Medicare Locals ceased operations on 30 June 2015, replaced by Primary Health Networks, an Australian Government initiative responsible for medical services in primary healthcare, secondary care and hospitals).

The PR component of the intervention was directed by the Medicare Local, as an adapted version of their healthy eating and lifestyle (HEAL) programme for chronic disease management and termed “Respiratory HEAL” for the purposes of differentiating from the standard programme. The programme was adapted to include the core facets of PR, as described in Lung Foundation Australia’s Pulmonary Rehabilitation Toolkit [16]; namely a pre and post programme assessment, two exercise sessions per week of at least an hour’s duration over a six to eight week course, and a disease education component. Thus, the Respiratory HEAL programme was conducted in the local community, as part of the public healthcare system and was available via general practitioner (GP) referral though Medicare as part of a GP “long consultation” and GP care plan. The programme was accessible to patients without a GP referral but incurred a cost to participants in this setting.

### Quantitative data

For eligible patients attending Respiratory Clinic, reasons for exclusion or non-participation were collected for quantitative summary. For participants, data were collected during the baseline visit and subsequent visits at five and ten months, including demographic information, review of social parameters, the results of detailed physical assessments, blood tests and questionnaire assessments of mental health status, lifestyle risk factors and COPD symptom burden.

### Post-intervention qualitative data

We explored the perspectives of the healthcare team, and patients. A respiratory specialist physician, a registered clinic nurse, a case coordinator, a GP and three patients (N = 7) agreed to participate in the interviews. Table 1 illustrates the composition of the healthcare team members, who contributed to the qualitative data and their roles within the TEAMcare project.

**Table 1:** Composition of TEAMcare: the TEAMcare providers who contributed to qualitative data

Healthcare professional	Gender	Description of Role
Respiratory Physician	Female	Assessment of COPD and comorbidities, preparation and review of COPD treatment plans
Registered Clinic Nurse	Female	Support assessments, administer aspects of treatment plan (eg smoking cessation counselling, instruction in inhaler technique, case coordination
Research Assistant	Female	Case coordination, data entry and management
General Practitioner	Male	Prepare health care plan, receive and integrate test results in between study visits, provide referrals to specialists, allied health and Respiratory HEAL

The qualitative interviews were undertaken following completion of the post-intervention quantitative data collection. A structured interview approach was used for the patient interviews (Additional file 3) based on previous research on the feasibility of integrated primary care [6], and a semi-structured interview approach was used for the health care provider interviews. To begin the semi-structured interviews, participants were asked their involvement in TEAMcare, and questions related to the feasibility, acceptability, facilitators and barriers to the multidisciplinary care intervention (TEAMcare). Subsequent questions were generated by the participants’ responses, and were also informed by the previously collected quantitative data. The interviews were individual face-to-face or telephone conversations, and ranged from 15 to 45 min in duration. All interviews were audio-recorded, transcribed verbatim and entered into NVivo 9. The data was analysed using qualitative thematic analysis to identify key concepts and themes [17]. Data analysis began with the aim of coding the interview text. A code (or node as described by QSR NVivo 9) is a label assigned to describe a sequence of words, most often a sentence or paragraph [17, 18]. Each of the texts were read and re-read and nodes were developed along the way. Text was coded into either a new or a previously created node. By comparing and contrasting the data obtained from the TEAMcare provider and patient interviews, the nodes were merged to form ‘tree nodes’ as common sub-themes, and themes emerged from the data [17, 18]. Qualitative research is a reflexive process and as such research team discussions were undertaken to ensure a balanced and accurate interpretation of the data [19].

## Results

### Quantitative data

Fifty COPD patients were assessed for inclusion in the study protocol and 12 participants were recruited. Of 30 patients excluded, seven were excluded twice, often for differing reasons. The commonest reasons for exclusion were, in order of frequency, acute exacerbation of COPD within the last six weeks 36 % (13/36), respiratory failure 22 % (8/36), current or recent participation in standard PR 17 % (6/36), unstable cardiovascular disease 11 % (4/36), participant in another COPD study 8 % (3/36), non-ambulant, extreme frailty/poor prognosis and the need to prioritise another medical issue 3 % (1/36 for each). For patients in whom more than one exclusion criterion existed, the one listed was that deemed most important. Of 12 patients who refused participation, three had been excluded on a previous occasion.

**Fig. 1:**
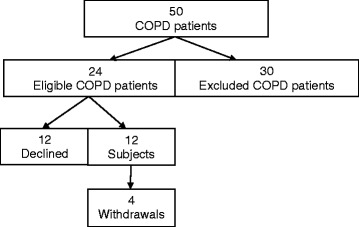
Recruitment

Of the twelve recruited participants, four withdrew and none completed the PR programme (Fig. 1). The main reasons for study withdrawal or non-participation related to: disinclination to attend PR 31 % (6/16), carer, work and other commitments 25 % (4/16) and the burden of increased appointments 12 % (4/16).

The study was terminated prematurely due to poor recruitment and clear lack of feasibility. After ten months’ recruitment, only 12 of 50 COPD patients were recruited to participate in the TEAMcare protocol, as for the majority of those excluded it was on the basis of safety criteria precluding participation in community-based PR. Moreover, the slow recruitment resulted in prolonged delay or non-enrolment in PR due to insufficient numbers to form a group for the sessions. In effect, the primary goals of the pilot study had been achieved at this point.

Enrolled participants were average age 64 years and mostly overweight (83 %), females (75 %) with significant smoking exposure (84 %, including 17 % current smokers). Lung function was moderately impaired with average FEV1/FVC ratio 0.53 and predicted FEV1 58 % and FVC 90 %, respectively. COPD assessment test (CAT) score of 10 or greater indicated significant COPD symptom burden in 67 %. Most were taking at least one long-acting bronchodilator medication and inhaled corticosteroid (92 % for each). Cardiovascular medication had been prescribed in 58 % and serum vitamin D levels were insufficient in 88 % (values <30 nmol/L and 30–60 nmol/L regarded as deficient and insufficient, respectively) (Table 2). COPD management plans for TEAMcare participants are provided in Table 3 to provide context for the qualitative data.

**Table 2:** Baseline characteristics of the participants in the TEAMcare for COPD pilot trial

Patients	N = 12
Mean age (years)	64 (11)
Gender (female)	9/12 (75 %)
Education level (High school or less)	8/12 (67 %)
Smoking status	
Current smoker	2/12 (17 %)
Former smoker	8/12 (67 %)
Moderate-Vigorous exercise (hours/week)_a_	2.5 (1.9)
Fruit serves/day (≥2)	6/12 (50 %)
Vegetable serves/day (≥4)	4/12 (33 %)
Unintentional weight loss (≥5 kg past 6 months)	2/12 (17 %)
Overweight (BMI 25–29.9)	3/12 (25 %)
Obese (BMI ≥30)	7/12 (58 %)
High waist circumference (≥102 cm men, ≥88 cm women)	9/12 (75 %)
High blood pressure (≥130/80 mmHg)	7/12 (58 %)
Mean FEV1% (SD)	58 (16)
Mean FVC% (SD)	90 (20)
Mean FEV1/FVC (SD)	0.53 (0.13)
Mean SpO_b_%	97 (1.7)
Inhaled medications	
LAMA	7/12 (58 %)
LABA	9/12 (92 %)
ICS	9/12 (92 %)
SAMA	1/12 (8 %)
Cardiovascular medications	7/12 (58 %)
Diabetes medications	2/12 (17 %)
Other medications	7/12 (17 %)
Impairment (CAT score ≥10)	8/12 (67 %)
Breathlessness (mMRC score Grade ≥2)	4/12 (33 %)
Clinically relevant depression (PHQ-9 score ≥10)	1/12 (8 %)
Clinically relevant anxiety (GAD-7 score ≥10)	2/12 (17 %)
Clinically relevant somatic symptoms (PHQ-15 score ≥10)	3/12 (25 %)
Blood tests	
White cell count (x 10^9/L)_b_	9.0 (4.7)
C-Reactive Protein (mg/L)_b_	8.5 (4.8)
25-hydroxyvitamin D (<60 nmol/L)	7/8 (88 %)

Legend: *BMI* body mass index, *FEV1* forced expiratory volume in one second, *FVC* forced vital capacity, *SpO*_*2*_ oxygen saturation estimate, *LAMA* long-acting antimuscarinic agent, *LABA* long-acting beta agonist, *ICS* inhaled corticosteroid, *CAT* COPD assessment test, *mMRC* modified Medical Research Council, *PHQ* patient health questionnaire, *GAD* general anxiety disorder

_a_ missing one observation; _b_ missing four observations

**Table 3:** TEAMcare patients’ structured management plans

Participant	Individualised management plan
Participant 2 (ACOS)	Initial Further assessment: glycaemic status, osteoporosis Goal: weight loss, symptom control 5 months Identified: impaired glycaemic control, dyslipidaemia Assessment: suboptimal symptom control Plan: change inh (device and regimen), repeat RFT, reconfirm lipid and glycaemic results, commence Resp HEAL
Participant 3 (COPD, OSA, T2DM, CRF, CCF)	Initial Goal: weight loss, recommence CPAP 5 months Assessment: deterioration in heart failure (addressed by cardiologist) Identified: elevated scores for anxiety and depression, suboptimal glycaemic control and dyslipidaemia (on treatment), stably impaired renal function Plan: further assessment/referral for anxiety and depression, reassess treatment for diabetes (dyslipidaemia within acceptable limits), extend duration of supplemental oxygen from nocturnal to 24 h/day, recommence CPAP, broach advanced directives/end of life care plan, check Pneumococcal vaccination status 10 months Assessment: Improved physically/psychologically, using CPAP Plan: change inh (drug), update COPD action plan, reconcile medications (GP), check Pneumococcal vaccination status
Participants 4 (ACOS, OSA, T2DM)	Initial Goal: weight loss Plan: improve inh adherence 5 months Identified: deterioration in anxiety, Plan: correct inh technique, further assessment of anxiety 10 months Plan: change inh regimen (drug and device), refer to psychologist (GP)
Participant 7 COPD, emphysema	Initial Goals: smoking cessation Further assessment: osteoporosis, glycaemic control Plan: NRT for smoking cessation, further assessment anxiety/depression 5 months Successfully quit smoking, anxiety/depression scores improved Plan: change inh regimen (drug and device), BMD scan, ENT review if dysphonia continues
Participant 8 COPD, ABPA, OSA, T2DM	Initial Further assessment: osteoporosis Goal: weight loss, symptom control Plan: check anti-pneumococcal antibodies 5 months Identified: osteopaenia, suboptimal glycaemic control, dyslipidaemia Plan: change inh regimen (drug), reassess lipids and glycaemic control post exercise programme, endocrinology review
Participant 11 COPD, OSA,	Initial Goal: weight loss, recommence/maintain CPAP Further assessment: osteoporosis Plan: monitor hypertension 5 months Identified: renal impairment Assessment: hypertension controlled with new antihypertensive Plan: commence Resp HEAL, adjust inh (reduce dose)
Participant 12 COPD	Initial Goal: weight loss Plan: change inh (drug and device), influenza and Pneumococcal vaccination, cardiology review 5 months Identified: resumed smoking, depressive symptoms, poor inhaler technique Plan: smoking cessation, further assessment of depression, change inh (drug and device)

Legend: *ACOS* asthma/COPD overlap syndrome, *OSA* obstructive sleep apnoea, *T2DM* type II diabetes mellitus, *CRF*, chronic renal failure, *CCF* congestive cardiac failure, *ABPA* allergic bronchopulmonary aspergillosis, *inh* inhaled treatment, *RFT* respiratory function tests, *Resp HEAL* respiratory HEAL programme, *CPAP* continuous positive airways pressure, *NRT* nicotine replacement therapy, *BMD* bone mineral density, *ENT* ear, nose and throat (surgeon)

### Qualitative data

Thematic analysis was applied to the qualitative data to identify key concepts and to inform subsequent initiatives. Themes identified included implementation challenges for both patients and healthcare providers. For the clinicians, issues identified pertained particularly to resources – staff time and balancing workload priorities, identifying a succinct, effective means of communication between hospital and community care providers and deciding on the most appropriate site (hospital outpatient clinic or general practice) from which to conduct the TEAMcare programme. Issues for patient participants included barriers related to motivation and functional impairment, access to transport, and resolving conflicting personal/professional priorities and commitments.

### TEAMcare - implementation challenges

#### Need for a case manager

The clinical team identified several problems implementing the TEAMcare model using existing resources within the public hospital system. TEAMcare was to have a dedicated case manager, envisaged to be a role undertaken by the clinic nurse. However, the health service allocated additional responsibilities to the clinic nurse, subsequent to commencement of the study.

*I am the bone density technician, I am the immunology nurse, the wound care nurse, I do the asthma clinic. I have a busy week. I have overload…I had my own patients and then the TEAMcare patients so I if I had a patient that wasn’t on TEAMcare I would be giving them less attention as I had so many things to do for the TEAMcare.*

The project’s objective was to remain within existing healthcare system resources and hence the existing nurse took on extra workload in data collection, the respiratory department’s research assistant took on the coordinator role and the respiratory physician covered the respiratory clinic nurse’s role during periods of extended leave.

*One of the envisaged things was to have a case manager … I think that would be an essential component for this to go forward. I wouldn’t contemplate doing something like this again unless we had increased staffing…I don’t think it is feasible without it.*

Leave cover for clinic nurse duties in Respiratory Clinic is not always available, which was problematic; the clinic mostly runs without a nurse during periods of leave, which presents a challenge for case coordination. It was argued by the healthcare team that a dedicated respiratory nurse, experienced in case management, is required for successful coordinated care.

The respiratory physician highlighted the difficulty in justifying a dedicated case manager for a programme such as TEAMcare:

*There is certainly evidence for PR but there isn’t robust evidence for case management…you need something that is quite definite and positive to convince the health administrators for extra staff.*

#### Coordinated care & GP burden

The respiratory physician postulated that the general practice setting, rather than the specialist setting, might have been the more suitable for care coordination because generally the GP is the hub of outpatient management and their role already encompasses coordination between multiple specialists and healthcare providers.

However, there was consensus amongst the hospital-based healthcare team that this would place substantial extra burden on the GP. In addition, it was deemed difficult for GPs to provide the coordinated care as the entire first visit for TEAMcare, including assessment and treatment plan formulation, took much longer than a standard consultation, since most of the participants had significant co-morbidities identified.

The case coordinator described her experiences attempting to facilitate the Respiratory HEAL programme referrals:

*It is the GP referring the patient to Medicare Local, that is the thing I don’t see working because they are very busy and they won’t help as we want them to…The GP would think what the hell is this, more paperwork for me, no. They would ask the secretary to fill out the form [referral] and fax it. So it is too much work for them.*

The GP representative commented that he did not find the extra workload to be problematic:

*When we do the TEAMcare treatment plan, we need to do a form, just a couple of pages (laughs), that’s all.*

However, he noted that he only had one patient involved with TEAMcare and that it may have been much more burdensome for him, had more patients been participating, because he cared for many COPD patients.

#### Communication with GPs

Communication between the TEAMcare hospital-based clinicians and the GPs was found to be problematic as described by a member of the healthcare team:

*I think the communication particularly with the GPs could have been better. We had it as a written communication with an explanation about the project…a summary of the assessment…and there would be a request for pulmonary rehabilitation…That was very clumsy…and that was a bit awkward as the GPs were not used to making referrals to pulmonary rehabilitation… There was a lot of chasing up about the pulmonary rehab referrals… So we ended up giving an instruction sheet with the fax number on it and partially completed for the GP.*

However, the GP representative was very positive about his experience and involvement with the TEAMcare programme.

*I think the TEAMcare programme was very good, it was good working with the allied health and hospital system and the patient did not have any out of pocket expense, and the patient liked it and we liked it. So it was good. TEAMcare is really helpful for the COPD patients…the respiratory nurse, there was a respiratory nurse for the breathing technique and breathing exercises.*

#### Patient engagement and health literacy

PR is integral to COPD management management guidelines and hence TEAMcare included PR as a central focus. This proved problematic for a number of reasons. The healthcare team reported that patients who refused to participate in the study, or failed to continue with the programme, held a negative perception of the exercises associated with PR, believing that they would be too vigorous or strenuous. Some believed that the exercises would cause distressing breathlessness or be detrimental to their health.

The respiratory nurse commented:

*People declined for different reasons. The main one was they didn’t want to do the PR.*

*They don’t see the bigger picture and some of them are not accountable…Well, if they have been a smoker…I do the smoking counselling and you see them with COPD but they don’t quite connect they have got COPD because of their lifestyle…it could have been this or that, parent’s smoking….*

The healthcare team stressed the need for patient education to highlight the importance and benefits of PR in order to engage patients in the process.

In summary, the key themes discussed in the interviews with regard to implementation challenges focussed on identifying the best site for coordinated COPD care, the workload for both hospital and community healthcare staff and negotiating effective communication around Medicare referral requirements. Persuading patients that PR was beneficial was identified as a particular challenge. Although only one GP contributed to the qualitative analysis from the perspective of providing healthcare in the community, and so the insights provided may not be representative, the discrepancy between hospital healthcare providers and the GP is notable in terms of the TEAMcare project. The GP’s comments about TEAMcare programme were overwhelmingly positive, citing no concerns about the workload related to extra referrals, including the Respiratory HEAL programme. However, an opinion about the feasibility of shifting TEAMcare into general practice, with GPs as central coordinators was not sought.

### TEAMcare participation

The patient participants who took partin TEAMcare generally expressed positive reactions, describing the programme as *more inclusive* and *thorough*. These participants also described how they were empowered with new knowledge from the programme:

*I have started to become to be aware on how to lose all this weight. For my nutrition, I basically um, learnt ways in recognising hidden sugars, so I was able to pay attention to hidden sugars, different labelling on products so I was able to be nutritional (laughs). I am able to recognise any changes in my health; I am able to manage my own medications.*

Some of these participants related the exercise as contributing to improving their lung function. One stated that *exercise is good for my asthma*…*gets you more active*. Another described how she had learnt to manage her exercise tolerance:

*Just exercising, like exercise tolerance being just little things like even getting up every half hour, even if it is just walking around the table, and moving and breathing differently*.

### TEAMcare Challenges for Patients

#### Motivation and Functional Impairment

A patient participant who had attended the PR described how she had learnt to maintain her motivation:

*I mainly learnt that you have got to keep going, you have got to get up, approach the day, you know, without thinking of ‘I don’t feel well’. There were a few days when I felt like that but in the main I get up every morning, I make sure I’m showered…I am quite happy about how it has turned out. I think it’s that old cliché, use it or lose it, that’s what they said when I went to exercises when I didn’t feel like it.*

She recognised the importance of tailoring the exercise programme to her level of capability:

*I didn’t go on the very heavy things, I went on the light things…but it has made me move forward.*

Another participant spoke about her ‘commitment’ to the TEAMcare programme:

*Sometimes it was a little difficult, but I made the commitment (PR).*

Many positive effects of PR programmes are attributed to the participation in regular, supervised exercise within the patients’ assessed capabilities, resulting in increased endurance and functional capacity. However, the beneficial contribution from other components, such as the social interaction and cooperation entailed in group activities, is less well understood. Regular reassurance or affirmation was deemed very important by several patients.

Because only few patients attended PR, several spoke about their desire for the PR to be ‘more social’. They reported that they often attended the PR by themselves, or with one or two others. A patient stated: *It is better if you are in with a group of people, and there weren’t many, I think it should be more advertised.*

The wide age range of PR attendees was seen as detrimental to some patients’ enjoyment and motivation. This may have been accentuated by the difficulties encountered introducing the modified PR programme, due to slow recruitment and the small absolute number of patients participating, as this phenomenon has not been described in the literature for conventional PR. The case co-ordinator commented:

*There was a big age difference…so they didn’t communicate and it was boring for them…. I don’t think anybody thought about that actually.*

Regular follow-up was deemed important to maintain motivation. However, the case coordinator described the difficulties experienced in trying to maintain the follow-up arrangements for the assessments and extra tests involved in the patients’ individualised management plans.

*The follow-up is very time intensive…patients forget to do things and then I had to call them up every single week, and I was asking them “have you gone to the GP”, and they would say, “no, no”. They would say, “I have this form but I haven’t looked at it”.*

#### Access to transport

Easy access to transport to attend PR was viewed as crucial by the healthcare team and patients. PR was held in a small district town approximately 15 km from the main hospital. Although the participants attended the Respiratory Outpatients’ Clinic at the same venue, an occasional visit to see the specialist was less burdensome than regular (twice weekly) visits for PR. The hospital-based clinical team identified this as a major hurdle for some patients:

*It was difficult to get there [PR] for some people, and um, most of them were older so they had to organise transport and it was difficult for them every week. The public transport is not good…to take the bus is a nightmare!*

#### Barriers of time, competing priorities and organisation

The respiratory nurse reported that patients found the multiple tests and referrals to be overwhelming.

*One of the problems was we had to request many things, blood tests, spirometry, referrals to cardiologist, dietician, allied health professionals, sometimes psychiatry or psychology…the patients had many forms, many things to do at the same time and makes them overwhelmed…a lot of patients are over 65 or 70 and it is too much for them. Also the waiting lists are very long, it is too much, it is too much.*

When patients had multiple competing priorities, their emphasis often differed markedly from that of the healthcare team. For example, patients tended to delay returning to their GP and, because a referral was required, this in turn delayed commencement of PR.

*Well, they needed at least six or eight people to start the programme. I was pushing them [the patients] to go to the GP for the referral and then we will start the programme, and they said “oh, no, I have to do another thing, I have another appointment”, so that delayed the start of the exercise programme sessions.*

The patients’ comments about the organisation and coordination of TEAMcare were largely positive but most commented on the delay to commencing PR.

*I found it [TEAMcare] to be very good…probably a little more inclusive, more thorough…although it was a bit disorganised to get started.*

Patients frequently had competing personal and professional priorities and commitments. These often took precedence. Some patients were employed, which prevented participation and, in a few instances, necessitated withdrawal from the study. In addition, PR was conducted during business hours, which meant that patients who maintained employment were unable to attend.

Patients’ own health needs would often be given secondary importance to other personal commitments as identified by a patient:

*My husband is retired…so we are going to walk around together but we haven’t started yet um, because we have had a lot of upsets with the son.*

## Discussion

The TEAMcare intervention outlined a number of implementation challenges for patients, healthcare professionals and organisations in the delivery of clinic-based, guideline-driven, multidisciplinary, team-based care for COPD patients.

Overall, the themes identified from qualitative analyses included ‘resource limitation’, ‘COPD patient health literacy’, ‘patient motivation’, ‘conflicting personal/professional priorities and commitments’. The TEAMcare intervention was viewed positively by the representatives for general practice and the participating patients. The hospital-based clinical providers were more circumspect, likely because they were directing the intervention and so were acutely aware of its implications for healthcare resources. The GP representative felt that it was good experience managing his COPD patient as part of a team, including allied health professionals and viewed positively the PR programme’s availability through Medicare. The patients viewed the programme as holistic and thorough and perceived benefits of the Respiratory HEAL programme to their lungs and general health.

The hospital-based healthcare team identified significant resource challenges to providing the TEAMcare collaborative COPD management intervention. They found that a case coordinator role was essential to the project, otherwise the patients became overwhelmed with the multiple referrals and appointments and failed to complete the enrolment process for PR. At present, the healthcare service provides no leave cover for the respiratory clinic nurse, which is problematic for continuity of the programme, since TEAMcare assessments require cooperation and contribution between both nursing and medical healthcare providers. They expressed concern that the GP’s role in TEAMcare would be burdensome to a practitioner known to have meagre time resources, although the GP representative did not perceive this as a problem. Whether subsequent interventions would work better in the general practice setting with the GP, or practice nurse, responsible for case coordination and management was not explored in our study but should be considered in future endeavours. A strong rationale for considering the GP setting for future COPD chronic management initiatives is the potential to access patients with less severe disease than seen in a specialist respiratory clinic. These patients are more appropriate to a community-based PR programme, which may not be able to provide supplemental oxygen or monitoring of oxygen saturations during exercise. In fact, the need to exclude from TEAMcare, patients with established respiratory failure requiring long term domiciliary oxygen therapy substantially limited recruitment in our study, which would not have been the case if PR was being delivered within the GP setting. If the specialist respiratory service is deemed the preferred location to house the collaborative team-based care model, then it would be important to deliver a PR service with capacity to provide increased monitoring and, potentially, supplemental oxygen to its population of COPD patients with more advanced disease and higher prevalence of respiratory failure. It would also be crucial to establish with local GPs their preferred means of communication. This study used written communication conveyed to the GP via the patient and although the GP representative was reasonably satisfied with the communication of the individualised patient management plan, the patients visited their GPs far less frequently than expected and this caused delays in activating the TEAMcare plans, especially with respect to PR referrals and enrolment. Presumably bolstering communication with a “back up” such as mailed or emailed plan information would improve the efficiency of plan uptake and action.

Our results are generally consistent with the medical literature on PR and on other coordinated care programme initiatives. Common themes include health literacy and patients’ reluctance to engage in PR [20] due to ignorance of its beneficial effects in COPD and fears about potential detrimental effects, particularly overexertion [21]. This leads to poor enrolment and completion statistics for PR programmes. Addressing this barrier is a priority for any effective team-based care programme in COPD and hence subsequent interventions should incorporate an educational component regarding PR [21]. One simple strategy worth considering is to provide educational material coupled with patients describing their own experiences in PR in the form of a brief video, such as “Introduction to Pulmonary Rehabilitation”[22], which could easily be shown to a “captive audience” waiting in Respiratory Clinic. The intervention needs to occur early in the process to ensure their commitment, so that the patients are proactively seeking this effective form of treatment, rather than missing their first PR assessment visit. Alternatives for the more technologically adept, include e-learning via podcast, websites etc.

Another problem to solve for our group, as for others working in this area, is access to PR programmes for patients in terms of service availability and waiting lists, competing priorities, especially for patients who work [21] and in terms of transport options (for the frail, impaired, elderly or oxygen dependent [20, 23]). In fact, attendees of the conventional hospital-based PR programme can utilise hospital transport for the journeys to and from the sessions, which is not available locally for any of the community-based programmes. Models such as telehealth or home PR bypass this issue [23], but potentially at the cost of social interaction, which was deemed important by our patients.

Options to improve the accessibility of PR will likely require funding commitment from the healthcare service, with potential to expand the hospital-based allied health staffing, expand and divide services to encompass more locations, including within the local community and expansion of patient transport options into the local community as well. The remaining consideration, which will be perhaps the greatest challenge, is provision of PR programmes for those COPD patients remaining in employment, who stand to benefit most from the intervention.

In summary, the implementation of a collaborative team-based COPD management intervention trialled in this pilot project was not feasible in its current form. Our pilot data suggest future full-scale research on team-based models of care for COPD management appears feasible in the hospital setting, although effective implementation requires strategies on facilitating: 1) COPD patient motivation and health literacy; 2) access to PR outside standard business hours; 3) better integration with hospital team providers and general practice; and 4) health resources for a task-dedicated case manager for enhancing treatment fidelity. We acknowledge that consideration of several study limitations is warranted. These include premature closure of the pilot, small sample size, saturation not achieved for the qualitative data and inability to reach trustworthy conclusions. However, our findings are consistent with those from a recent systematic review of relevant studies, including several in patients with COPD, on factors influencing the implementation of Chronic Care Models in primary care settings [24].

## Conclusions

Delivering structured, multidisciplinary care, including PR, to COPD patients presents challenges. This pilot data provides valuable insights to inform future design and implementation initiatives. In addition to enhancing health resources, to engage COPD patients, a focus on health literacy and improving health service access, may be required.

## Additional files

Additional file 1: TEAMcare sequence. (PDF 73 kb) Additional file 2: TEAMcare review sequence. (PDF 66 kb) Additional file 3: Post-intervention structured interview questions for patient participants. (PDF 13 kb)
