# Supplementary material for: Implementation challenges in delivering team-based care (‘TEAMcare’) for patients with chronic obstructive pulmonary disease in a public hospital setting: a mixed methods approach
Source: BMC Health Serv Res. 2016 Aug 3;16:347. doi: 10.1186/s12913-016-1592-2 (PMC4973053; doi:10.1186/s12913-016-1592-2)
Supplement: Additional file 1: — TEAMcare sequence. (PDF 73 kb) [file 12913_2016_1592_MOESM1_ESM.pdf]

TEAMcare Sequence

Eligibility: Moderate to severe COPD – symptoms and spirometry criteria

1. >40 years □
2. Symptoms consistent with COPD (dyspnoea, cough or wheeze)□
3. Post bronchodilator spirometry: COPD ≥GOLD stage II □ (FEV1/FVC<70%, FEV1 <80%)
    a. Session quality (A or B):_________________
    b. FEV1 (number, %predicted):_____________
    c. FVC (number, % predicted):______________
    d. FEV1/FVC:____________

Exclusions:

1. Unable to walk □
2. Currently enrolled in pulmonary rehab program □
3. Not proficient communicating in English
4. Resp failure PO2<60, SpO2<88%, qualifies for home oxygen □
5. AECOPD within 6 weeks □
6. Unstable cardiac disease within 8 weeks (angina, uncontrolled heart failure, hypo or hypertension, tachy or bradyarrhythmia)□
    a. SBP<100, SBP >180 or DBP>100
    b. HR >100 or HR<50
7. Poor prognosis for survival, acutely unwell □
8. Routine observations
    a. BP_______________
    b. HR________________
    c. RR________________
    d. SpO2______________

Study Flow:

Clinic nurse

1. Date
2. DOB
3. Age (years)
4. MRN
5. Gender
    a. male =1
    b. female=2
6. Height
7. Weight
8. Waist Circumference
9. BMI
10. Smoking status
    a. current smoker □ smoking cessation counselling □
    b. former smoker□
    c. never smoker□

    d. exhaled CO□

11. Inhaled medications
    a. Drug, device, strength, daily dose
    b. Drug, device, strength, daily dose
    c. Drug, device, strength, daily dose
    d. Drug, device, strength, daily dose
12. Inhaler technique assessed □
13. Inhaler technique corrected □
14. CAT score □

<u>Enrolment (BC or EM):</u>

Subject info sheet □

Consent□

<u>Study data</u>

15. Ethnicity_________________
16. Education level (highest achieved)_________________
17. Occupation status___________________
18. Relationship status___________________
19. Parental status______________________
20. Living arrangements_____________________
21. GAD7 □
22. PHQ 9 □
23. PHQ 15 □
24. Lifestyle questionnaire
25. MMRC dyspnoea scale

Respiratory Specialist assessment:

1. If smoker, pharmacotherapy for nicotine dependence offered □
2. Formal lung function □
3. Review COPD medications □
    a. inhaler technique, if new device□
4. Vaccinations
    a. Flu □
    b. pneumococcal □
5. Oxygen assessment □
6. EOLCP □
7. Bloods (FBE, CRP, ESR, UEC, TFT, vit D, Ca, Mg, PO4, Hba1c, fasting glc and lipid profile, SHBG, total testosterone/oestrodiol)□
8. Bone density (if meets criteria) □
9. Medical comorbidites
    a. Depression or anxiety □ further psych assessment needed □
    b. CAD, heart failure, cor pulmonale □ cardiology assessment needed □
    c. Osteoporosis □ further assessment needed □
    d. Diabetes/glucose intolerance □ further assessment needed □
    e. Hyperlipidaemia □ further assessment needed□

    f. Other (list)

10. PRP/HEAL referral documents □
11. GP info sheet □ and letter □
