# Supplementary material for: Implementation challenges in delivering team-based care (‘TEAMcare’) for patients with chronic obstructive pulmonary disease in a public hospital setting: a mixed methods approach
Source: BMC Health Serv Res. 2016 Aug 3;16:347. doi: 10.1186/s12913-016-1592-2 (PMC4973053; doi:10.1186/s12913-016-1592-2)
Supplement: Additional file 2: — TEAMcare review sequence. (PDF 66 kb) [file 12913_2016_1592_MOESM2_ESM.pdf]

TEAMcare review sequence

Week 10 □ Month 5 □ Month 10□

1. Routine observations/assessment
    a. BP________________
    b. HR_________________
    c. RR_________________
    d. SpO2_______________
2. Adverse outcomes
    a. AECOPD □
    b. Death □
    c. Other □ specify:_____________________

Study Flow:

Clinic nurse

1. Date____________________
2. MRN____________________
3. Height(cm)___________________
4. Weight(kg)___________________
5. Waist Circumference(cm)_____________________
6. BMI_______________________________
7. Smoking status
    a. current smoker □ smoking cessation counselling □
    b. exhaled CO□
8. Inhaled medications
    a. Drug, device, strength, daily dose________________________
    b. Drug, device, strength, daily dose
    c. Drug, device, strength, daily dose
    d. Drug, device, strength, daily dose
9. Spirometry if regimen changed□
10. Inhaler technique assessed □
11. Inhaler technique corrected □
12. CAT score □
13. Other medications
14. Occupation status
    a. Changed□
    b. Unchanged□
15. Relationship status
    a. Changed□
    b. Unchanged□
16. Living arrangements
    a. Changed□
    b. Unchanged□
17. GAD7 □

18. PHQ 9 □
19. PHQ 15 □
20. Lifestyle questionnaire□
21. MMRC dyspnoea scale□

Respiratory Specialist assessment:

1. If smoker, pharmacotherapy for nicotine dependence offered □
2. Review COPD medications □
    a. inhaler technique, if new device□
3. Vaccinations
    a. Flu □
    b. pneumococcal □
4. Oxygen assessment □
5. COPD action plan □
6. EOLCP □
7. Bloods (FBE, CRP, ESR) □
8. Check progress medical comorbidites
    a. Depression or anxiety □ further psych assessment needed □
    b. CAD, heart failure, cor pulmonale □ cardiology assessment needed □
    c. Osteoporosis □ further assessment needed □
    d. Diabetes/glucose intolerance □ further assessment needed □
    e. Hyperlipidaemia □ further assessment needed□
