# Supplementary material for: Implementation challenges in delivering team-based care (‘TEAMcare’) for patients with chronic obstructive pulmonary disease in a public hospital setting: a mixed methods approach
Source: BMC Health Serv Res. 2016 Aug 3;16:347. doi: 10.1186/s12913-016-1592-2 (PMC4973053; doi:10.1186/s12913-016-1592-2)
Supplement: Additional file 3: — Post-intervention structured interview questions for patient participants. (PDF 13 kb) [file 12913_2016_1592_MOESM3_ESM.pdf]

Design and delivery

- What did you think about the elements of TEAMcare (like/dislikes)?
- Did you clearly understand the roles and tasks among TEAMcare members?
- How would you describe your interactions with TEAMcare members?
- Can you comment of the quality and level of contact with TEAMcare members?
- How would you describe the organisation and coordination of TEAMcare?

Self-management support

- Can you describe your role, as explained by TEAMcare members, in managing your health?
- What types of self-management support strategies did you learn from TEAMcare?
- What types of TEAMcare and community resources were organised to support you self-manage your condition?
- What health-related information did the TEAMcare members share with you to encourage your participation?

Results

- In your opinion, what have been the overall results of this TEAMcare program?
- Could tell us what have been both the most successful and the most problematic elements in this TEAMcare program?
- We have now covered all the main points we are interested in. Do you have any general observations or conclusions about this TEAMcare project that you would like to tell me now?
